# Implantable Cardioverter Defibrillator Tachycardia Therapies: Past, Present and Future Directions

**DOI:** 10.3390/jcdd11030092

**Published:** 2024-03-20

**Authors:** Andrew M. Leong, Ahran D. Arnold, Zachary I. Whinnett

**Affiliations:** National Heart and Lung Institute, Imperial College London, London SW3 6LY, UK; ahran.arnold@imperial.ac.uk (A.D.A.); z.whinnett@imperial.ac.uk (Z.I.W.)

**Keywords:** defibrillator, implantable cardioverter defibrillator, ICD, anti-tachycardia pacing, CIED, arrhythmia risk stratification, primary prevention, AI, competing risk, ventricular arrhythmia, ventricular fibrillation, ventricular tachycardia, sudden cardiac death

## Abstract

Implantable cardioverter defibrillators (ICDs) have a long history and have progressed significantly since the 1980s. They have become an essential part of the prevention of sudden cardiac death, with a proven survival benefit in selected patient groups. However, with more recent trials and with the introduction of contemporary heart failure therapy, there is a renewed interest and new questions regarding the role of a primary prevention ICD, especially in patients with heart failure of non-ischaemic aetiology. This review looks at the history and evolution of ICDs, appraises the traditional evidence for ICDs and looks at issues relating to patient selection, risk stratification, competing risk, future directions and a proposed contemporary ICD decision framework.

## 1. A Brief History of the Implantable Defibrillator

Losing his mentor to an arrhythmic sudden cardiac event, it has been more than 50 years since Dr Michel Mirowski conceptualized the automatic defibrillator [1]. Unhindered by the scepticism of his peers, Mirowski believed in the potential of such a device, and that defibrillating within the heart would require less energy, allowing one to construct a smaller capacitor that could be implanted. The first prototype of an internal-external defibrillator was created by 1972 [2], and efficacy with low energy defibrillation was demonstrated successfully in terminating ischaemia-induced ventricular fibrillation (VF) in 9 out of 11 patients undergoing coronary artery bypass grafting (CABG) [3].

On the 4th of February 1980, Dr Levi Watkins performed the first human implant on a 57-year-old lady with recurrent episodes of ventricular fibrillation (VF) remotely after a myocardial infarction, at the Johns Hopkins Hospital. A defibrillator electrode coil was placed in the superior vena cava (SVC) and a flexible patch electrode was attached epicardially over the apex via a left thoracotomy. The generator was positioned subcutaneously in an abdominal pouch. She would go on to develop VF which would be successfully terminated by defibrillation [4]. These early defibrillators were not programmable and were designed to recognise and terminate VF only. They were massive by today’s standards, weighing 280 g and 170 mL in volume.

In 1987, anti-tachycardia pacing (ATP) was demonstrated by Lindsay et al. to successfully terminate monomorphic ventricular tachycardia (VT) in 22 patients [5]. The Endotak system was introduced in 1988, allowing for a fully transvenous system, obviating the need for a thoracotomy and permitting a pectoral placement of the generator [6].

By the early 1990s, second and third generation ICDs had progressed to offer more effective tachycardia discrimination algorithms, increased programmability, ATP and bradycardia pacing. In addition, the development of biphasic waveform shock delivery and the use of the pectoral generator can as an electrode resulted in significant reductions in the defibrillation thresholds (DFTs) [7].

## 2. Supporting Evidence for ICD Use

### 2.1. Secondary Prevention

ICD indications are traditionally classified as either primary or secondary prevention. Secondary prevention indications were established by three landmark trials that were carried out largely in the 1990s—AVID, CIDS and CASH [8,9,10]. AVID [8] investigated 1016 patients with resuscitated VF, sustained VT with syncope or sustained VT with an LV ejection fraction (LVEF) < 40% and haemodynamic compromise. Patients were randomized to ICD or usual medical therapy, which comprised primarily amiodarone. Patients in the ICD arm experienced an overall greater survival at 1 to 3 years follow-up. The CIDS trial [9] included 659 patients, with documented VF, out of hospital cardiac arrest requiring defibrillation or sustained VT with an LVEF < 35% causing presyncope, who were randomized to ICD or amiodarone therapy. The primary outcome of all-cause mortality was non-significantly lower in the ICD group at 3 years. Finally, the CASH trial [10] studied 288 patients with resuscitated cardiac arrest secondary to documented ventricular arrhythmia (VA) with no reversible cause. They were randomized to ICD or anti-arrhythmic drugs. At the mean follow-up of 57 months, there was a non-significant reduction in all-cause mortality in the ICD arm. The majority of patients recruited into these studies had ischaemic heart disease, but each study contained approximately 10–15% non-ischaemic cardiomyopathy patients. A meta-analysis [11] of the three trials showed the ICD conferred a 28% all-cause mortality reduction over the medical therapy group, predominantly due to a reduction in arrhythmic death.

### 2.2. Primary Prevention in Ischaemic Cardiomyopathy (ICMP)

In ischaemic cardiomyopathy, the role of a prophylactic ICD was studied in several trials. Of note, MADIT-1, MUSTT, MADIT-II and SCD-HeFT (which was a mixed ischaemic and non-ischaemic cardiomyopathy trial) were of significance. MADIT-1 [12] investigated patients with prior MI and an LVEF ≤ 35% with NSVT and inducible VT. A total of 196 patients were randomized to ICD or anti-arrhythmic drugs, and at 27 months follow-up, there was a 54% reduction in mortality seen in the ICD arm. MUSTT [13] was not designed to evaluate the efficacy of ICDs, but rather to compare an electrophysiology study (EPS)-guided approach to a standard medical therapy approach. A total of 704 patients with prior myocardial infarction (MI), an LVEF ≤ 40% and NSVT were studied. Patients were randomized to medical therapy or an EPS guided therapy arm. In the EPS guided arm, if VT was inducible, patients could receive an ICD if at least one anti-arrhythmic drug was ineffective. At 5 years of follow-up, the primary end point of arrhythmic death or resuscitated sudden cardiac death (SCD) and the secondary end point of all-cause mortality was lower in the EPS guided group, primarily driven by patients receiving an ICD. A second analysis of MUSTT [14] suggested that, in the LVEF ≥ 30% subgroup, inducible VT on EPS might predict arrhythmic death. MADIT-II [15] simplified the inclusion criteria, studying 1232 patients with prior MI at least 30 days prior and LVEF ≤ 30%. They were randomized to ICD or medical therapy. The trial was terminated early after a mean follow-up of 20 months, in view of better survival in the ICD arm, where all-cause mortality was 35% lower than in the medical therapy arm. SCD-HeFT [16] recruited 2521 patients with New York Heart Association (NYHA) II or III heart failure for at least 3 months and an LVEF ≤ 35%. They were randomized to placebo, amiodarone or ICD. All patients received standard heart failure therapy, which comprised a beta-blocker and ACE inhibitor at the time. Of note, 48% of patients had cardiomyopathy of a non-ischaemic aetiology. All-cause mortality at 5 years was significantly reduced by 23% in the ICD arm when compared to placebo, and seen in both ischaemic and non-ischaemic cardiomyopathies. Long--term follow-up data of SCD-HeFT were recently published, and showed that the survival benefit persisted at 11 years [17].

Two large trials studying the role of a primary prevention ICD against standard care in the early post-MI period failed to show a survival benefit. DINAMIT [18] studied 674 patients with a recent MI (within 6 to 40 days), LVEF ≤ 35%, depressed heart-rate variability and elevated average heart rate on Holter ≥ 80 bpm. The exclusion criteria included acute or NYHA IV heart failure, three vessel PCI or planned CABG and VT occurring > 48 h after MI. Patients were randomized to ICD vs. no ICD. At a mean follow-up of 30 months, there was no significant difference in the primary outcome of mortality between the arms. Similarly, IRIS [19] randomized 898 patients with a recent MI (within 5–31 days), with either a reduced LVEF ≤ 40% and resting heart rate ≥ 90 bpm, or NSVT ≥ 150 bpm. At a mean follow-up of 37 months, there was no difference in the primary outcome of all-cause mortality.

Finally, the benefits seen in the RCTs have translated into the real-world setting as shown in two large prospective registries [20,21], where a 27% relative risk reduction in all-cause mortality was demonstrated.

### 2.3. Primary Prevention in Non-Ischaemic Cardiomyopathy (NICMP)

The role of a primary prevention ICD in patients with NICMP has come under more scrutiny since the publication of the DANISH trial [22] in 2016.

AMIOVIRT [23] was an early trial that randomized 103 patients, with NICMP with an LVEF of ≤35% and asymptomatic non-sustained ventricular tachycardia (NSVT), to ICD or amiodarone. There was no difference in the primary end point of all-cause mortality at 1 or 3 years. The CAT trial [24] enrolled 104 patients with NICMP of recent onset and an LVEF ≤ 30%, and randomized them to ICD or standard therapy; the study was terminated early because the all-cause mortality did not reach the expect 30% at 1 year.

Following the early small and negative trials, the DEFINITE trial [25] enrolled 458 patients with NICMP with an LVEF ≤ 35% and NSVT or >10 premature ventricular complexes (PVCs) per hour on Holter monitoring, and randomized participants to ICD or medical therapy alone. At 29 months of follow-up, all-cause mortality was non-significantly lower in the ICD group (HR 0.65, CI 0.4–1.06) with statistically significantly fewer sudden deaths in the ICD group (HR 0.2, CI 0.06–0.71). The all-cause mortality rate was lower than expected and may have led to underpowering of the study to detect a benefit of ICD therapy.

In SCD-HeFT [16], which was covered earlier, 48% of patients had NICMP. At 5 years, there was a 23% mortality reduction in the ICD group, and this benefit was consistent in both the ischaemic and non-ischaemic groups.

The COMPANION trial [26] enrolled non-ischaemic cardiomyopathy patients with an LVEF ≤ 35% and NYHA III or IV symptoms with a recent hospitalization in the last year. They were randomized to three groups—cardiac resynchronization therapy-defibrillator (CRT-D), cardiac resynchronization-pacemaker (CRT-P) and standard care in a 2:2:1 fashion. All patients received optimal pharmacological therapy of angiotensin-converting enzyme inhibitors (ACEi) or angiotensin receptor blockers (ARBs), beta blockade and spironolactone as tolerated. A total of 1520 patients were enrolled, and the CRT-D and CRT-P arms achieved a 34–40% reduction in the composite primary outcome of all-cause mortality or all-cause hospitalization compared with standard care. Only the CRT-D arm achieved a statistically significant reduction in all-cause mortality of 36% compared with the standard care group at 1 year.

The data supporting a primary prevention ICD in NICMP patients were, at that stage, largely from subgroup analyses. In addition, significant advances were made in medical therapy for HFrEF as well as the introduction of CRT. This was the context for the design of the DANISH trial [22]. A total of 1116 patients with symptomatic heart failure and NICMP with an LVEF ≤ 35% were randomized to receive an ICD or standard treatment. At 5.6 years of follow-up, there was no significant difference in the primary outcome of all-cause mortality between the two groups. There was, however, an absolute risk reduction of 3% in arrhythmic death, which was partially offset by a 1.5% absolute risk of device infection. This finding was unchanged at 9.5 years of follow-up [27]. The mortality rate in the trial was low, probably owing to the utilisation of modern heart failure therapies—ACEi or ARBs (97%), betablockers (92%), mineralocorticoid receptor antagonists (MRA) (58%) and CRT (58%). A subgroup analysis revealed that younger patients (<59 years of age) appeared to derive a survival benefit. There was a high rate of non-cardiovascular mortality in the trial (31%), suggesting that competing risk factors for mortality were high in the study population, influencing the all-cause mortality outcome.

**Figure 1:**
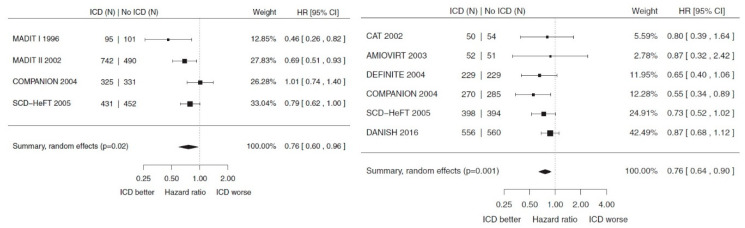
Meta-analysis of relevant primary prevention trials in ICMP and NICMP patients, demonstrating an all-cause mortality benefit when compared with standard therapy.

Several meta-analyses subsequently took place, pooling data from the five trials (CAT, AMIOVIRT, DEFINITE, SCD-HeFT and COMPANION) and the DANISH trial [28,29,30,31,32]. They showed that, when taken together, in NICMP patients, a prophylactic ICD conferred a 19–24% mortality reduction when compared with standard care. When only CRT patients from COMPANION and DANISH were analysed, there was a non-significant trend toward survival benefit in CRT-D over CRT-P. Figure 1 illustrates the findings of the meta-analysis by Shun-Shin et al. [28].

### 2.4. ICD Use in Other Clinical Settings

There are other clinical settings in which ICD use may be beneficial. Patients with inherited cardiomyopathies such as hypertrophic cardiomyopathy (HCM) and arrhythmogenic cardiomyopathy (ACM), and cardiac ion channel diseases, such as long QT syndrome (LQTS) and Brugada syndrome (BrS) [33], are at increased risk of life-threatening ventricular arrhythmias. While there is general agreement that survivors of an arrhythmic cardiac arrest should be offered an ICD for secondary prevention [34,35,36,37], the use of primary prevention devices in patients with primary electrical diseases has been less well studied. In the absence of large, randomized control trials, consensus opinion guidance is based on evidence from smaller trials [37,38]. It is recognised that arrhythmic risk varies in patients with inherited cardiomyopathies, for example, not all patients with HCM will experience a life-threatening ventricular arrhythmia. Risk is not static and may change with progressive phenotypic expression. Methods for risk stratification are, therefore, required to identify people at increased risk, in whom the benefits of ICD implantation are likely to outweigh the potential risks. This risk stratification may need to be performed on a regular basis [39]. The effects of treatment may alter the arrhythmic risk and therefore, need to be incorporated into risk stratification for primary prevention [39].

When considering a prophylactic ICD in HCM, two main risk stratification strategies are available: the first is based on the 2014 European Society of Cardiology (ESC) risk score and the second the 2020 American Heart Association (AHA)/American College of Cardiology (ACC) guidelines [36,38]. There are multiple variables in common between the two strategies, and a possible foreseeable addition might be genetic status for pathogenic or likely pathogenic sarcomere gene mutations [40].

The guidelines-recommended approach to primary prevention in ACM considers factors such as syncope, severe RV or LV systolic dysfunction, or moderate RV or LV systolic dysfunction with NSVT or inducible VT and the presence of certain genetic mutations [41]. Promisingly, Tourigny et al. developed a five-year ARVC risk calculator based on retrospective cohort data (which can be found at arvcrisk.com accessed on 1 January 2024), and identified seven key variables—sex, age, recurrent syncope, number of leads with T-wave inversions, 24 h ventricular ectopic count, NSVT and RVEF [42]. The calculator has been validated in multiple small studies [43,44,45,46] and, more recently, in an independent retrospective cohort of 429 patients from 29 centres in North America and Europe [47]. Another study suggested that the ARVC calculator had better performance in gene-positive ARVC than in gene-elusive patients [48]. The calculator is useful due to good discriminative performance but was noted to overestimate individualized risk in one of the studies [47].

The evidence pertaining to primary prevention in cardiac ion channel diseases like BrS and LQTS is even more complex and nuanced. In BrS, patients with documented sustained VT, arrhythmic syncope and, with less certainty, those with inducible VF during programmed extra stimulations using up to two extra stimuli, may be considered for a primary prevention ICD based on international consensus guidelines [37,49]. For patients with LQTS, guidelines suggest consideration for prophylactic ICD in patients with arrhythmic syncope or haemodynamically non-tolerated VA while on medical therapy or in those whom medical therapy is contraindicated or not tolerated [37,49]. In asymptomatic but high-risk patients (guided by LQTS type or based on the 1-2-3 LQTS risk calculator [50]), international guidelines suggest that prophylactic ICD may be considered [37,49].

Patients with advanced heart failure have high rates of ventricular arrhythmias and mortality [51]. A large retrospective observational trial demonstrated an immediate and sustained survival benefit of primary and secondary prevention ICD use in end-stage heart failure patients awaiting transplant [52]. However, the data supporting ICD use in patients on left ventricular assist devices (LVADs) are less definite and mainly from observational and registry cohorts. A significant proportion of these patients develop VAs [53] that are often well tolerated due to the preservation of cardiac output by LVADs; however, the effect on survival is not clear. Based on observational data on older-generation pulsatile LVADs, ICD use did appear to be associated with longer survival [54,55,56]. However, the effect of ICD use on survival in newer generation continuous-flow LVADs is not consistent [57,58,59]. Furthermore, the INTERMACS registry data of patients on continuous-flow LVADs did not show an association of ICD use with survival benefit [53].

## 3. Risk vs. Benefit of ICDs

The dilemma we face when deciding whether to recommend ICD implantation to our patients is whether the benefit of having the device implanted is likely to outweigh the costs (risk of complications and healthcare costs). There are several factors that influence this risk–benefit balance, which can be broadly divided into factors that increase the chance that a device will deliver benefit (life-saving treatment of ventricular arrhythmias) vs. factors that reduce the cost of the device. The ideal scenario would be that we could reliably identify all people who will develop a life-threating ventricular arrhythmia and implant an ICD before they develop this arrhythmia and that ICDs have a very low cost (of complications, inconvenience and monetary cost). Since we are some way off from achieving both these objectives, we currently have to weigh up the risks vs. benefits.

### 3.1. Patient Selection

The aim of ICD implantation is to extend life by treating ventricular arrhythmias and thereby preventing sudden arrhythmic death. When selecting patients, the objective is to identify persons who are at sufficiently high risk of dying as a result of sudden arrhythmic death that the benefit of having an ICD outweigh the current costs of having an ICD implanted (risk of complications and financials).

To demonstrate a reduction in all-cause mortality with ICD implantation in a clinical trial requires a sufficiently high-risk population to be investigated (i.e., enough life-threatening ventricular arrhythmia events need to occur during the study duration). For example, if the event rate is very low, a very large sample size and/or follow-up duration would be required to demonstrate benefit. If we were to target a low-risk population, device costs would need to be very low, since the numbers needed to treat them would be very high.

The second important consideration when considering a reduction in all-cause mortality is the competing risk of death. To illustrate this—if a patient has a high risk of dying due to a non-arrhythmic cause, even if they have the substrate for a ventricular arrhythmia, then they are unlikely to live long enough for a ventricular arrhythmia event to shorten their life.

Reliably quantifying the risk of sudden arrhythmic death remains a challenge, and there is a need for the development of more sophisticated methods for calculating risk. Left ventricular ejection fraction is a widely used method for identifying people at risk in the primary prevention population, but has its limitations, both in terms of specificity (a significant proportion of patients with reduced ejection fraction never experience a life threatening arrythmia) and sensitivity—as the Myerburg paradox illustrates, in terms of absolute numbers, most sudden cardiac deaths occur in patients with an LVEF > 35%.

As such, a major anticipated change would be developing more sophisticated methods for quantifying the risk of developing life-threatening arrhythmias. As our understanding of the genetics and the associated clinical phenotypes of inherited arrhythmia syndromes advance, together with new scoring tools and AI models, the identification of higher-risk patients will be made earlier. Lamin cardiomyopathy is an example where the weight of a genetic diagnosis strongly influences a primary prevention ICD indication outside the standard LVEF criteria [37].

A ventricular scar provides a substrate for the development of ventricular arrhythmias. An assessment of the presence of a ventricular scar has therefore been investigated as a method for identifying patients at risk of developing ventricular arrhythmias with promising results. Cardiac magnetic resonance (CMR) late gadolinium enhancement (LGE) allows for the quantification and characterisation of myocardial fibrosis. In NICMP, the prognostic value of CMR LGE was shown in multiple studies and a meta-analysis of 4554 patients [60], with the presence of LGE demonstrating an odds ratio for cardiovascular mortality of 3.4 and for VA an odds ratio of 4.52 when compared to the absence of LGE. In a retrospective study of 979 patients with coronary artery disease and a wide range of LVEFs [61], Zegard et al. studied the association of VAs and SCD with CMR LGE in the form of any myocardial fibrosis on visual assessment (MF_VA_) and a quantified cut-off of grey zone myocardial fibrosis (GZF), which was theorized to be a substrate for VAs. They demonstrated that MF_VA_ and GZF were superior predictors of sudden cardiac death and VA than LVEF as shown in diagnostic accuracy statistics and multivariate analyses. It was found that the absence of any myocardial fibrosis was associated with no SCD or VAs, suggesting that the LGE had a 100% sensitivity for SCD or VA, compared with only 57% when taking LVEF ≤35% as a cutoff. In addition, there was a strong net reclassification improvement, which has promising clinical applications in patient selection and avoidance of unnecessary ICD implants. In a prospective study of 700 patients with ICMP and NICMP undergoing ICD or CRT-D implants, MF_VA_ was strongly associated with SCD when compared to the absence of it (HR 26.3), with GZF adding incremental predictive value. In addition, MF_VA_ had a negative predictive value of 100% at up to seven years of follow-up [62]. Prospective randomized trials will need to take place to provide more robust evidence to support its use in clinical practice.

In addition to CMR LGE, CMR diffusion tensor imaging (DTI) is an emerging technique that promises to reveal incremental and complementary scar characterization, without the need for gadolinium-based contrast [63].

A future study [64] aims to assess the role of a prophylactic ICD in patients with mild to moderate LV systolic impairment (LVEF 36–50%) and a CMR LGE scar. Another ambitious study [65] aims to determine if an ICD will have a benefit in patients with an LVEF range of 36–50% in the presence of some combination of non-invasive risk factors, including clinical history, imaging findings (including CMR LGE), PVCs and NSVT, SAECG late potentials, QTc, T-wave alternans, heart rate variability and heart rate deceleration. Two trials will evaluate whether CRT-D will confer any additional benefit over CRT-P, in all chronic heart failure patients on optimal medical therapy and with a CRT indication [66] and in NICMP patients without significant CMR LGE [67].

A proof-of-concept paper employing a novel ultra-high-frequency ECG assessment of QRS complexes in inherited arrhythmia syndromes compared with healthy subjects found an association between multiple high-frequency peaks, which represent QRS fragmentation (even if none were seen on a standard ECG) and arrhythmia risk [68]. In a small retrospective study of NICMP patients undergoing EP study for risk stratification, electrical restitution-based ECG markers (regional restitution instability index and peak electrocardiogram restitution slope) of ventricular arrhythmia were associated with VA or death in these patients [69].

It is conceivable that AI will play a significant role in improved arrhythmia risk stratification as well. As early as 2019, Attia et al. demonstrated that by applying AI-enhanced ECG (AI-ECG) to a single ECG recording in sinus rhythm, they were able to predict paroxysmal AF with an AUC of 0.87 [70]. A systematic review and exploratory metanalysis of 46 studies that used electrophysiological signals to predict malignant VAs concluded that the AI models developed achieved a high performance, and that there is potential for the personalized prediction of malignant VA, although significant methodical limitations were identified in multiple studies [71]. AI-ECG applied to heart failure patients was found to be more discriminatory than conventional methods in determining SCD, especially in patients with an LVEF range of 35–50% and NICMP, and was able to discriminate between SCD and non-SCD [72].

Conversely, the application of AI may assist us in identifying patients who would not benefit from an ICD and hence avoid an invasive and largely permanent procedure. A machine learning model incorporating clinical and ECG data trained on a large historical cohort of ICD patients was able to predict the absence of arrhythmic mortality at three years in an independent cohort with robust performance [73].

AI techniques have been applied to imaging as well. Applying ML to LGE-CMR scars in a cohort of stable CAD patients, an AI model was able to outperform traditional guideline-based clinical and LVEF criteria in predicting arrhythmia [74].

In the future, it would be desirable to utilise more reliable risk stratification models, incorporating clinical, genetic, electrical and imaging data, to generate an absolute risk reduction and absolute harm statistic to assist in ICD decision making.

### 3.2. Competing Risk

The original ICD trials recognised the issue of competing risk and attempted to mitigate this by excluding people with an expected prognosis of less than 1 year, and those with advanced heart failure who were at high risk of mortality from pump failure. In the future, it would be pertinent to develop more sophisticated methods of quantifying competing risk, which might include other measures of risk. A large, controlled cohort study of 2327 patients with prophylactic ICD implants, from 44 cities in 15 European countries, suggested that older patients and diabetics derived less survival advantage [20]. DILEMMA [75] is an ongoing randomized controlled trial designed to address the questions of prophylactic ICD use in the elderly. The trial will recruit 730 patients, 75 years or older, with primary prevention ICD indications, to determine if there is a benefit of a prophylactic ICD.

End-stage renal failure patients on dialysis were systematically excluded from all landmark trials, and there are no specific recommendations made for this group of patients in international society guidelines. A retrospective propensity-matched study in end-stage renal failure patients on dialysis did not find an association between prophylactic ICD and survival [76]. A pooled analysis of chronic kidney disease (CKD) patients who underwent CRT implantation from the MADIT-CRT and RAID trial showed that the patients with more advanced CKD experienced a high non-arrhythmic mortality rate and a lower rate of ventricular arrhythmias, and did not appear to attain the same degree of benefit from a primary prevention ICD [77]. A retrospective analysis of 3535 diabetic patients in the EU-CERT-ICD registry [78] and a large meta-analysis of 162,780 patients, comparing diabetic and non-diabetic patients with ICDs [79], suggested that, as a group, they received attenuated or no benefit from prophylactic ICDs. Prospective randomized data in these groups of patients are lacking.

Sex-related differences are yet another consideration. Women, as a group, are underrepresented in the major clinical trials and there are data to suggest that women with heart failure have a better overall prognosis than men, and experience less sudden cardiac death [80,81,82]. A meta-analysis of the major non-ischaemic cardiomyopathy prophylactic ICD trials, pooling results for female and male participants separately, suggested that women, when compared with men, may not experience a significant benefit from prophylactic ICDs in this setting [83].

The Seattle Proportional Risk Model (SPRM) and MADIT-ICD score are currently available scores that have been developed to better estimate competing risks [84,85,86]. Both scoring systems utilise clinical variables, including age, body-mass index, LVEF, NYHA score, blood pressure, serum sodium and creatinine, digoxin levels, diabetes status, resting heart rate, NSVT, IHD and use of CRT. The MADIT-ICD score achieved a C-index of 0.67 when tested on an external cohort [86]. A multimodal machine learning model, leveraging ECG time-series features, on top of traditional clinical variables, showed incremental discrimination when compared with a MADIT-ICD score on an external cohort [73]. Further validation of these scoring systems in prospective and contemporary ICD cohorts is needed to ensure generalizability to real-world primary prevention candidates.

Further developments in quantification of competing risk would prove valuable when developing more sophisticated methods of patient selection.

### 3.3. Contemporary HFrEF Therapy

A further consideration with risk quantification is that the risk associated with a particular risk factor does not necessarily remain static over time. This is relevant to the landmark trials, which proved the benefit of primary prevention ICDs in patients’ heart failure. Since those trials were conducted, new medical therapies for heart failure have emerged with the potential to alter arrhythmic risk. In particular, both angiotensin receptor/neprilysin inhibitors (ARNIs) and sodium-glucose cotransporter 2 inhibitors (SGLT2is) have been demonstrated to improve overall outcomes in heart failure. More recently, the glucagon-like peptide-1 receptor agonist Semaglutide was shown to improve cardiovascular outcomes and survival in certain groups of overweight patients [87,88,89]. In the SELECT trial, about one quarter of study patients had diagnosed heart failure (almost a third of which reduced ejection fractions); however, more research is needed, specifically addressing HFrEF patients and arrhythmic outcomes.

Entresto was shown to reduce sudden cardiac death in the PARADIGM-HF trial [90]. In a cohort of HFrEF patients with existing ICDs or CRT-Ds, the initiation of ARNI resulted in less ventricular arrhythmias and ICD interventions [91]. A recent meta-analysis of 8837 patients from four randomized and non-randomized trials of HFrEF patients showed that the ARNI was associated with a significant reduction in sustained VT, NSVT, ICD shocks and improved biventricular pacing [92]. Despite favourable prognostic outcomes with SGLT2i in HFrEF, there did not appear to be a significant effect on ventricular arrhythmias [93,94].

A recent independent meta-analysis evaluating the theoretical effect of ICD implantation on patients treated with SGLT2i and ARNI showed that these new agents did not affect the mortality benefit of the prophylactic ICD [95].

The guidelines recommend a primary prevention ICD if the LVEF fails to improve beyond 35% after ≥3 months of optimal medical therapy, likely reflecting a compromise between uncertainty about the timeframe for LV remodeling with medical therapy against the unprotected arrhythmic risk in these patients. Emerging data suggest that LV remodeling may lead to LVEF improvements beyond 3 months, stretching as far as 12 months [96,97]. To illustrate this, in PROVE-HF [98], patients with primary prevention ICD indications at baseline were initiated on ARNI on top of optimal medical therapy, and 32% improved their LVEF beyond 35% at 6 months, and 62% by 12 months. In light of these data, authors have suggested that guideline recommendations be re-evaluated [99].

The potential change in baseline risk with contemporary heart failure medication has led several investigators to perform trials to re-evaluate indications for ICD implantation.

These studies should be designed with adequate power to detect a difference in survival in a contemporary cohort with modern HFrEF therapies with lower expected mortality rates, which may have been one of the pitfalls of the DANISH study. The BRITISH study aims to enroll 2500 patients with NICMP with an LVEF ≤35% and assess whether the use of CMR-defined scar to direct ICD implantation is associated with a reduction in mortality [100]. The CMR-ICD study will enroll 760 patients with NICMP with an LVEF ≤35% and CMR LGE scar and assess if there is a benefit to an ICD in that population [101]. Taking personalized therapy a step further, the SPANISH-1 study will recruit 900 patients with NICMP and an LVEF ≤ 35%, and use pathogenic or likely pathogenic genetic variants on top of CMR LGE in deciding on the need for a primary prevention ICD [102]. PROFID is a large European collaboration study, evaluating post-myocardial infarction patients with symptomatic heart failure and an LVEF ≤ 35%, to determine if drug therapy alone is non-inferior to drug therapy and prophylactic ICD in terms of benefit and harm [103]; in addition, exploratory variables will be studied, including CMR LGE, genomics and AI-ECG.

An apt take-home message is the need for individualized patient selection based on life expectancy, the patient’s values, quality of life and comorbidities, which are reflected in contemporary guidelines [37,104]. In addition, the likely reduced absolute benefit of prophylactic ICDs in the contemporary HFrEF patient must be re-evaluated against the acute and chronic risks associated with a long-term implantable device when selecting patients.

## 4. ICD Cost Reduction

Reducing the cost of having an ICD implanted is the other side of the risk–benefit balance. The less intrusive and safer an ICD becomes the more acceptable ICD implantation becomes, potentially even in lower risk populations.

Since they were initially developed, ICDs have undergone significant refinement in almost every aspect. Superficially, the weight of a modern device has been reduced to approximately 70–90 g, with a volume of around 30 millilitres. Device longevity has improved significantly with the development of LiMnO_2_ and hybrid LiSVO/CFx batteries. Manufacturers’ product performance reports suggest up to 13–17 years under specified conditions. This has reduced complications and costs, and improved quality of life for patients, however specific device reliability remains a significant consideration [105].

### 4.1. Shock Reduction

#### 4.1.1. Inappropriate Shocks

Inappropriate shocks are a significant barrier to patients having an ICD implanted. Having an inappropriate shock causes patients significant psychological harm [106] and older studies suggested that inappropriate shocks are associated with a 1.5- to 2-fold increase in mortality [107,108,109,110]. A more recent analysis [111] that combined data from five landmark trials (MADIT II, MADIT-Risk, MADIT-CRT, MADIT-RIT, RAID) found that only the first appropriate shock was associated with an increased risk of death. Inappropriate shock, inappropriate ATP and appropriate ATP were not independently associated with increased mortality. Appropriate shock for a VT ≥ 200 bpm and VF were the associated with the highest risk of death, approximately 3× higher than that of patients receiving no therapies, suggesting that the risk of death is related to the underlying substrate rather than the shock treatment itself. An appropriate ICD shock, particularly for a VT ≥ 200 bpm, should alert clinicians to a deterioration in heart failure and that substrate modifying treatment like catheter ablation should be considered [112].

Inappropriate shocks remain an important problem. Patients may decide that the risk of inappropriate therapies outweighs the potential benefits of ICD implantation and decide not to have an ICD implanted or choose to have therapies deactivated.

The causes of inappropriate shocks can be broadly divided into three main groups, namely: misdiagnosis of supraventricular arrhythmias (including atrial fibrillation and atrial flutter), cardiac oversensing and non-cardiac oversensing. Cardiac oversensing can occur as a result of T-wave, P-wave or R-wave overcounting. Non-cardiac oversensing can result from lead failure with noise or myopotentials.

ICD manufacturers have made progress in reducing the frequency of inappropriate shocks. From applying the product density function (analysing the amount of signal time in the isoelectric line) to diagnose ventricular arrhythmias in 1976, sophisticated tachycardia discrimination algorithms have now been developed to diagnose VAs and minimize the risk of inappropriate shocks, with all modern ICDs utilising some combination of rate, AV dissociation, A > V, morphology, interval stability and onset criteria.

**Figure 2:**
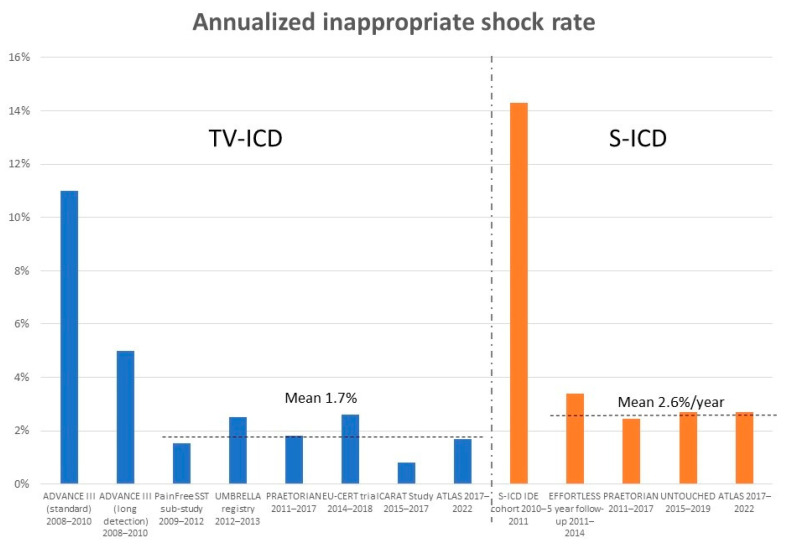
Annualized inappropriate shock rates from historical and contemporary sources.

These developments have resulted in a reduction in inappropriate shocks rates in contemporary studies compared with historical cohorts (summarised in Figure 2).

The CARAT study was a prospective, international, post-market study of 2052 MicroPort transvenous ICD and CRT-D systems implanted from 2015–2017, and demonstrated an inappropriate shock rate of 1.6% in 2 years [113]. This contrasts with the PRAETORIAN trial [114], where during a median follow-up of 49.1 months, 7.3% of transvenous-ICD (TV-ICD) patients received an inappropriate shock, of which 93% were from SVT/AF and the remaining 7% from oversensing, while 9.7% of subcutaneous-ICD (S-ICD) experienced an inappropriate shock over the same period. With the exception of PRAETORIAN and EFFORTLESS, which had 4–5 years of follow-up data, the remaining studies have relatively short follow-up periods of around 1–2 years. This is relevant as it has been shown that lead failures (a cause of inappropriate shocks) rise progressively with time after implantation [115] and in the same study the lead failure rate reached ~20% at 10 years. This figure is close to that found in a large insurance database cohort with devices implanted between 2003–2015, which showed a lead failure rate of 25% in 10 years [116]. A retrospective cohort of 2410 patients, between 2002 and 2014, demonstrated an annualized lead failure of 0.6%/year and demonstrated over an average follow-up of 3.9 years [117].

Despite the reduction compared to historical studies, the mean annualized inappropriate shock rates remain higher than ideal, with the annual rate of inappropriate shocks of 1.73% for TV-ICDs and 2.6% for S-ICDs, which translates into a 5-year projected risk of 8.7% and 13%, respectively. This is likely to be a conservative estimate as clinical trial patients tend to be better managed, arrhythmia tends to accumulate over time and leads would tend to fail later as discussed above.

**Figure 3:**
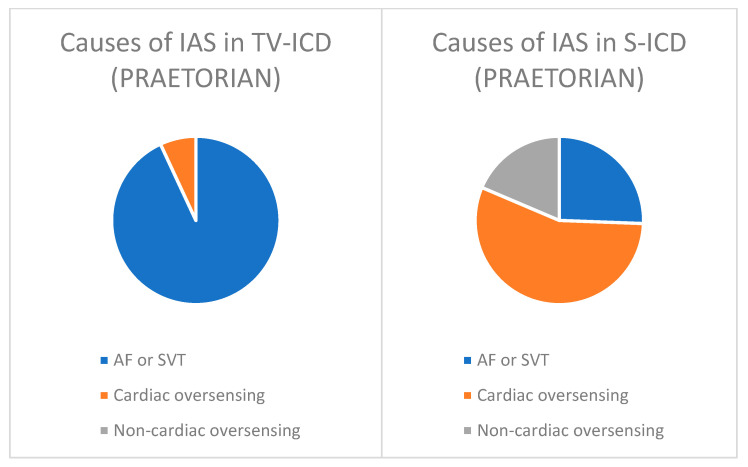
Causes of inappropriate shocks (IAS) in TV-ICDs and S-ICDs based on the PRAETORIAN trial data.

The causes of inappropriate shocks differ between transvenous and subcutaneous ICD. In the TV-ICD arm of the PRAETORIAN trial, 93% of inappropriate shocks were attributable atrial fibrillation (AF) and supraventricular tachycardias (SVT), while the remaining 7% were due to cardiac oversensing and there were none resulting from non-cardiac oversensing. Conversely, in the S-ICD arm, 25% were due to AF or SVT, 56% due to cardiac oversensing and 19% due to non-cardiac oversensing (Figure 3).

#### 4.1.2. Delaying Therapies

Despite being highly effective, the adverse associations with “appropriate shocks” were recognised in the mid to late 2000s, where data suggested an association with heart failure and mortality [18,108,118,119]. A pooled analysis of 2135 ICD patients from four major trials incorporating ATP to reduce shocks showed that shocked VA resulted in a 20% increase in mortality while ATP-treated arrhythmias did not have a similar association with increased mortality [120]. It was recognised that a significant proportion of VTs would spontaneously terminate, and that slower, well-tolerated VAs may not need to be shocked. This was supported by two non-randomized trials [121,122], followed by three randomized trials [123,124,125], which consistently demonstrated that programming a longer detection interval for therapy reduced unnecessary therapy with no compromise in safety. Metanalyses of these trials showed a mortality benefit with no increase in risk of syncope [126,127]. Similarly, a study focusing on secondary prevention patients showed that setting a high-rate cut-off and a long detection interval reduced appropriate and inappropriate therapies without increasing the incidence of syncope and slow VT [128]. On the other hand, case reports have shown undiagnosed slow VT manifesting as heart failure with worsening LV impairment and respiratory failure [129], as well as acute liver failure [130]. However, owing to the relatively small proportion of slow VT cases, around 6% [131], more research is needed on the optimal management of these patients who are often unrecognised or misdiagnosed [132] and would otherwise receive no device therapy unless VAs accelerate into the therapy zones or degenerate into VF.

#### 4.1.3. ATP

Unlike defibrillator shocks, ATP is painless and has fewer detrimental effects on the myocardium. The main adverse outcome of ATP is tachycardia acceleration. Initially intended as a treatment for slower VTs, an early study demonstrated efficacy in terminating 90% of VT with cycle lengths (CL) > 300 ms, with an acceleration in tachycardia of 2–4% [133]. Subsequently, a randomized controlled trial comparing ATP vs. shocks as first treatments for fast VT (188–250 bpm) demonstrated 81% successful termination of fast VT (CL < 320 ms) with similar rates of syncope and sudden cardiac death [134]. At the time, it was believed that the scar-based re-entrant VT in ischaemic cardiomyopathy would be more susceptible to ATP compared with a non-ischaemic substrate, but a metanalysis of 6127 patients from three large clinical trials showed that VA rates and ATP success rates were comparable in both aetiologies [135].

For the past two decades, there have been no significant advancements in the delivery of ATP. Conventional ATP takes the form of bursts, which are a train of impulses at a constant interval below the tachycardia cycle length, or ramps, which are a train of impulses with decrementing interstimulus intervals. Other than the type of sequence, adjustments to the number of sequences, number and amplitude of pulses, coupling and pacing cycles and the site of stimulus (e.g., delivery of ATP through LV lead) can be made.

A promising recent development is intrinsic ATP (iATP), which is present in the latest generation of Medtronic ICDs and CRT-Ds. It is a novel automated algorithm that utilises tachycardia CL and post-pacing intervals from a failed ATP sequence to optimise subsequent ATP sequences. Firstly, the number of S1 impulses is optimized based on the propagation time or pacing electrode-to-VT circuit time. When the VT circuit is reached, an S2 impulse is then delivered to advance the VT circuit and close the excitable gap. The S1–S2 interval is intended to be as short as possible without loss of capture, and the initial S2 is delivered just beyond the predicted myocardial refractory period and decrements by 20–30 ms with each unsuccessful attempt until loss of capture. After loss of capture, the S1–S2 interval is restored to the shortest interval with capture. Finally, an S3 impulse is added and decremented until success or a minimal coupling interval of 160 ms. Virtual modelling demonstrated a 17% increase in successful termination of VT when compared with conventional burst ATP with a similar acceleration rate [136]. The initial feasibility and safety trial of iATP demonstrated an approximately 80% cumulative success rate of cardioverting VT with iATP [137]; however, there are no published clinical trials to date comparing this with conventional ATP. A few case reports have described the success of iATP in VTs that were refractory to conventional ATP, and in 1 case, ATP and amiodarone therapy [138,139,140].

#### 4.1.4. Other Considerations for Reducing Therapies and VAs

Concurrent developments in catheter ablation for VT, AF, SVT and AV nodal ablations, remote monitoring [141,142] and medical therapy [90,143] have been shown to reduce the risk of inappropriate shocks as well. In addition to discriminating SVT, algorithms now exist to ameliorate the effect of R-wave double counting and T-wave oversensing [144], as well as the detection of specific lead failures with resultant oversensing (Abott SecureSense, Medtronic Lead Integrity Alert) [145,146,147]. Conventional CRT was shown to reduce appropriate ventricular arrhythmias and ICD shocks, particularly in responders [148,149,150]. In addition, left bundle branch area pacing CRT was associated with significantly fewer ventricular arrhythmias than conventional CRT in a recently published propensity score-matched analysis of 1778 patients [151], which may be due to the normalization of repolarisation with conduction system pacing [152].

#### 4.1.5. Failure to Treat Life-Threatening VAs

Minimizing unnecessary therapies must be balanced against the antithetical scenario of life-saving therapy being withheld for life-threatening VA. VF may manifest with low and erratic signal amplitudes and morphologies resulting in undersensing and/or falling below detection rates, and consequently evading conventional ICD detection algorithms. A case series of normally functioning ICDs that failed to deliver therapy for VF [153] showed that VF not satisfying programmed detection criteria occurred in 90% of cases. A prospective autopsy study of sudden deaths showed that 6.4% of ICD deaths occurred due to failure of VF therapy [154]. Using simulation on recorded VA intracardiac electrograms (EGMs), a novel algorithm was able to diagnose 81.9% of otherwise underdiagnosed cases of VAs [155]. This small but significant group of patients represents a blind spot and more study is needed to guide optimal management.

### 4.2. S-ICD

Since the introduction of the EndoTak system, the vast majority of ICDs have been transvenous, owing to their ease of implantation, in addition to efficient and effective low-energy therapies. However, associated acute and chronic complications are not insignificant. At implant, for example, even in experienced hands, the complications rate can be as high as 2.5–5%. These include lead-related central vein injury leading to a massive haemothorax, cardiac perforation resulting in tamponade, vascular thrombosis and embolisms [15,16]. Chronic complications are particularly relevant to young patients, namely structural damage, lead failure and infection warranting lead extraction, which is a complex procedure with serious complications such as vessel injury, a massive haemothorax, valve damage, cardiac perforation, tamponade and death [156,157].

To address some of these limitations, a fully subcutaneous ICD was developed in 2010, comprising a pulse generator and a single 3 mm lead. The pulse generator is implanted subcutaneously in a lower left lateral or posterior left lateral position in an intermuscular pocket between the serratus anterior and latissimus dorsi. Through a second xiphoid incision, the distal portion of the lead is tunnelled vertically along the left sternal midline and the proximal portion tunnelled along the sixth rib to the lateral axillary pocket where it is attached to the pulse generator.

Since then, multiple clinical trials assessing safety and efficacy have been published. A recent meta-analysis of 13 studies, comprising 9073 patients, demonstrated that S-ICDs are at least as effective and safe as TV-ICD for preventing sudden cardia death in patients without an indication for pacing [158]. The ATLAS trial [159] was the first superiority trial and demonstrated a 92% reduction in lead-related complications for S-ICDs compared with TV-ICDs at 6 months. In spite of this, the inherent limitations for S-ICDs remain, namely, bradycardia pacing, cardiac resynchronization and ATP. In addition, device longevity is a consideration, with real-world registry data suggesting 5–6 years [160,161].

A recent ongoing development is that of a subcutaneous ICD with a substernal lead (Extravascular ICD) [162]. In theory, closer proximity to the heart allows for ATP and short-duration bradycardia pacing to be administered. In addition, defibrillation energy requirements are lower, allowing for better device longevity and a more compact form factor comparable to modern TV-ICD generators (77 g, 33 mL). Early quality of life outcomes appear to be favourable when compared with S-ICDs and TV-ICDs [163]. Reliable defibrillation and low implant complication rates have been seen in early studies [162,164] thus far, but further study is needed to guide clinical practice.

Another technology designed to circumvent the lack of ATP in the S-ICD is the modular cardiac rhythm management system [165], which involves pairing an S-ICD system with a leadless single chamber pacemaker. The S-ICD uses unidirectional conductive communication to command the leadless pacemaker to deliver ATP [165]. Early pre-clinical studies have demonstrated feasibility in canine subjects [166]. This is being evaluated in the ongoing modular ATP study [167].

With a significantly lower lead-related complication rate, indications for extravascular ICD systems are likely to expand in the next decade, as the issues of battery longevity, ATP, pacing and cardiac resynchronization are gradually overcome, especially since some of these technologies are already undergoing clinical trials.

## 5. The Future

### 5.1. Reducing Inappropriate and Unnecessary Therapies

As described above, inappropriate shocks rates have improved to approximately 1.7–2.6%/year (8.7–13% over 5 years) in shorter follow-up studies but remain a major consideration and complication of ICDs. Current algorithms are entirely EGM-based and will thus have inherent limitations. A potential solution would be combining EGM with haemodynamic data. Using laser doppler perfusion monitoring and coupling it to EGM signals, an algorithm was able to distinguish between VF and artifacts that ICDs may interpret as VF, namely simulated lead fracture, EGM double counting and sinus tachycardia [168]. A haemodynamic sensor, in theory, would also be able to identify poorly perfusing slower VTs, and might be able to guide therapy below the traditional rate detection cutoffs. Yet another novel solution to assessing “haemodynamics” would be confirmation by mobile application [169]: a mobile application could warn a patient that a shock or ATP would be delivered within a prespecified time interval unless a button is clicked.

Due to the nature of EGM data, it is plausible that AI-enhanced EGM-based algorithms would also improve inappropriate shock discrimination. In the era of advances in physiological pacing, AV node ablation may be used increasingly to avert the issue of inappropriate shocks from SVT discrimination limitations, without compromising atrioventricular and interventricular synchrony.

### 5.2. New Delivery Technology

In addition to iATP as discussed above, the selection of the ideal target site for ATP will need further study. Applying ATP to the conduction system has theoretical benefits. Proximity of pacing site has been shown to affect the efficacy of ATP [170,171], and ATP into the conduction system could theoretically shorten the propagation time to the VT circuit; however, decrementation within the conduction system itself may be a consideration as well. A study in a canine heart model using ischaemia-reperfusion injury as a substrate for VT demonstrated that ATP applied to the His bundle resulted in less VT acceleration and VF than conventional right ventricular ATP, which may be explained by a more complete and organized capture of the myocardium [172]. Another study demonstrated that during VT episodes, synchronized atrial and conduction system pacing above the VT rate was able to improve haemodynamic stability and in some cases terminate the VT, although this was not the pre-specified goal of the study [173].

Medtronic has developed a low profile 4.7Fr defibrillator lead (LEADR ICD Lead) based on the SelectSecure 3830 lead well known for its use in conduction system pacing. The lead is lumenless and delivered by catheter, not unlike the 3830. It may provide greater reliability and allow placement in more challenging and smaller anatomies, in addition to reducing chronic vascular complications and tricuspid regurgitation, which is one of the weaknesses of bulkier defibrillator leads and the use of multiple leads. An ongoing study is taking place to assess the safety and efficacy of such a design [174]. A standard defibrillator lead was successfully implanted in the left bundle branch area (LBBa) in five patients, achieving LBBa pacing and successful DFT testing [175]. The LEADR ICD lead will likely be able to be deployed to a conduction system position as well—allowing for a single low-profile lead solution to cardiac resynchronization and defibrillation, with potential to apply ATP directly into the conduction system.

## 6. Conclusions

**Figure 4:**
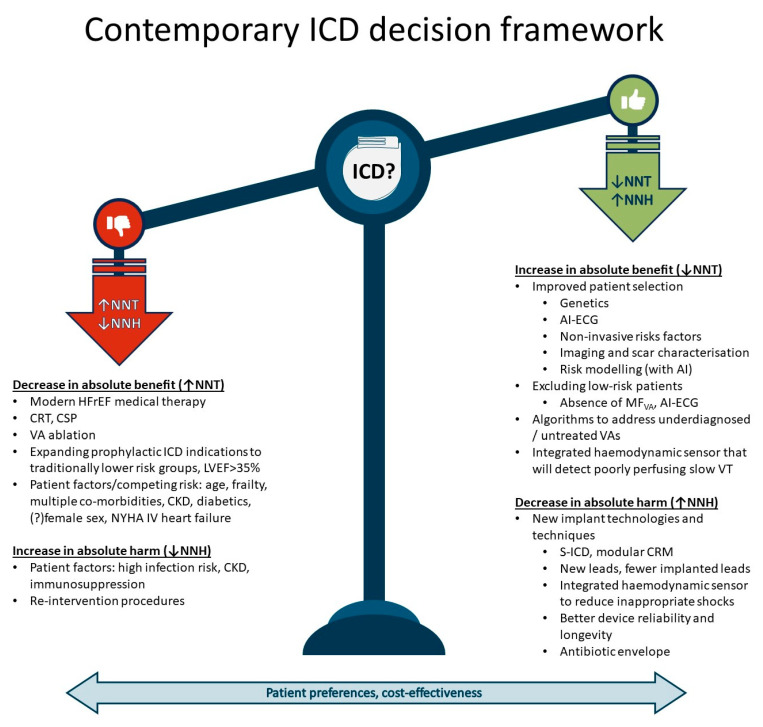
Main illustration—contemporary ICD decision framework.

The international guidelines appear deceptively simple when prescribing a clear decision-making matrix for the selection of patients for ICDs. However, the decision to implant an ICD has always been complex, especially so for a primary prevention device. With the rapid progress in the field of cardiology in the last decade, and the expectation of more significant changes to come, the physician has to weigh the absolute benefits against the absolute risks, with imperfect data, all while considering cost-effectiveness and the patient’s preferences. Modern HFrEF therapy, CRT, CSP and VA ablations have all likely increased the numbers needed to treat (NNT) to avoid a sudden cardiac death, while better patient selection will likely decrease NNT. New device technologies, and algorithms to better optimize therapy decision making, will likely increase the numbers needed to harm (NNH). As the absolute risk of implanting a device decreases, a prophylactic device will become more and more favourable for a larger population of patients with a possibly higher NNT. At this stage, cost-effectiveness will become an increasingly important consideration. The main illustration attempts to provide a framework of the variables that influence absolute benefit and absolute harm in contemporary practice and in the future (Figure 4).

The ICD remains a crucial and very relevant therapy in the cardiologists’ armamentarium. The physician must keep abreast of the latest evidence in this evolving field, as many changes can be expected in the coming decades.

## Data Availability

Not applicable.
